# Effectiveness of Meniscal Repair in the over-50 Population: Clinical Results with a Mean 2-Year Follow-Up

**DOI:** 10.3390/jcm15145491

**Published:** 2026-07-13

**Authors:** Michelangelo Delmedico, Simone Sanfilippo, Cleber Garcia Parra, Francesco Poggioli, Federico Chiodini, Giorgio Zappalà

**Affiliations:** 1 Orthopedics and Traumatology Department, ASST Papa Giovanni XXIII, 24127 Bergamo, Italy; cleber.garciaparra@gmail.com (C.G.P.); francesco.poggioli@gmail.com (F.P.); fchiodini@asst-pg23.it (F.C.); giorgio.zappala@libero.it (G.Z.); 2 Orthopedics and Traumatology Department, Cardinal Massaia Hospital, 14100 Asti, Italy; 3 Orthopaedics and Traumatology Department, CTO Hospital, 10126 Torino, Italy

**Keywords:** meniscal repair, meniscus suture, over 50, arthroscopic surgery

## Abstract

**Background/Objectives:** The purpose of this study was to evaluate the outcomes of meniscal suture repair in patients over 50 years of age with a minimum follow-up of 6 months, following failure of at least 6 months of conservative treatment. **Methods:** We retrospectively reviewed patients older than 50 years treated between 1 January 2017 and 30 June 2023 at the Orthopaedics and Traumatology Division of ASST Papa Giovanni XXIII (Bergamo, Italy) who underwent meniscal repair after failed conservative management. Patients underwent clinical, radiological and functional assessments. Meniscal healing was evaluated by MRI at 6 months postoperatively. Patient-reported outcome measures (PROMs), including KOOS, IKDC, Lysholm and Tegner Activity Scale, were collected preoperatively and at final follow-up. **Results:** Eleven patients were included, with a mean age of 53.4 ± 4.1 years (range, 50–64). Nine patients had lateral meniscus tears, and two had medial meniscus tears. At a mean follow-up of 22.0 ± 16.8 months (range, 6–66), significant improvements were observed in KOOS, IKDC and Lysholm scores (*p* < 0.05), with mean postoperative values of 95.4 ± 5.1, 87.5 ± 8.3 and 97.1 ± 3.5, respectively. All patients returned to their pre-injury activity level according to the Tegner scale. One complete failure and one partial failure were observed, both in patients with medial meniscus tears. **Conclusions:** In this small cohort, arthroscopic meniscal repair in patients over 50 years of age was associated with significant improvements in clinical and functional outcomes, with a failure rate of 18.2%. These findings suggest that meniscal repair may be a viable joint-preserving option in carefully selected middle-aged patients, particularly in lateral meniscus lesions. However, results should be interpreted cautiously given the limited sample size and retrospective design.

## 1. Introduction

Meniscal tears are the most prevalent sports-related injuries, with an estimated annual incidence of 60 per 100,000 individuals [1]. Notably, the medial meniscus is involved in approximately 75% of these cases [2]. Menisci withstand various forces, including shear, tension, and compression; play a crucial role in load transmission by converting vertical compressive forces into horizontal hoop stresses; cover 60% of the tibial plateau and transmit over 50% of the total axial load applied to the knee joint [3,4,5,6].

The ESSKA European Meniscus Consensus Group distinguishes between “meniscus tears,” which result from acute knee injuries, and “meniscus lesions,” which are degenerative and occur without a history of trauma [2,7]. Common types of meniscal tears include longitudinal (including bucket-handle) and radial tears, which are frequently associated with ligamentous injuries [8]. Degenerative meniscal lesions typically present as horizontal cleavages [7]. Their prevalence increases with age, ranging from 16% in the knees of women aged 50–59 to over 50% in men aged 70–90 [9].

Meniscal tears increase axial load transmission to the tibial plateau, posing a risk for osteochondral damage and arthritic degeneration [10]. Surgical treatment options include arthroscopic partial meniscectomy and meniscal repair using various suture techniques (all-inside, outside-in, or inside-out). Evidence suggests that patients undergoing total meniscectomy are at a higher risk for subsequent femoral condylar flattening and joint space narrowing, which lead to degeneration [11,12]. Recent trends indicate a shift toward meniscal suture repair, as supported by the 2019 ESSKA consensus, which asserts that a patient’s age does not significantly affect the failure rate of traumatic meniscus tears. However, degenerative changes in meniscal tissue may influence healing following suture [13,14,15]. The purpose of this study was to evaluate the effectiveness of meniscal suture repair in patients over 50 years of age who presented with clinical and radiological evidence of meniscal tears after failing at least six months of conservative treatment.

## 2. Materials and Methods

### 2.1. Study Design and Patient Selection

This retrospective cohort study was conducted in accordance with the ethical principles of the Declaration of Helsinki. Informed consent was obtained from all participants for inclusion in the institutional research registry. A retrospective chart review was performed to identify patients aged 50 years or older who underwent arthroscopic meniscal repair at the Orthopaedics and Traumatology Division of ASST Papa Giovanni XXIII, Bergamo, between 1 January 2017 and 30 June 2023. All patients had previously undergone a standardized six-month course of nonoperative treatment without satisfactory symptom resolution. This protocol included two intra-articular corticosteroid injections (40 mg triamcinolone acetonide combined with 1% lidocaine), a structured physiotherapy program focused on quadriceps strengthening and restoration of knee range of motion while avoiding weight-bearing hyperflexion for three months and oral non-steroidal anti-inflammatory drugs (NSAIDs) as needed. Surgical treatment was indicated in patients who continued to experience mechanical symptoms, such as locking, catching, or giving way, and/or recurrent knee effusion despite completion of the conservative treatment protocol.

Exclusion criteria were strictly applied as follows:

- Concomitant ligamentous laxity or injuries requiring reconstruction (e.g., anterior or posterior cruciate ligament tears);
- Advanced knee osteoarthritis, defined as Kellgren–Lawrence (KL) grade 3 or 4 on standard weight-bearing anteroposterior, lateral and Rosenberg views;
- Previous ipsilateral knee surgeries or subsequent conversion to arthroscopic partial meniscectomy during the designated conservative treatment window.

### 2.2. Preoperative and Postoperative Evaluation

The preoperative diagnostic workup included a comprehensive clinical examination, standard bilateral weight-bearing radiographs, long-leg standing radiographs, anteroposterior, lateral and Rosenberg views. In addition, all patients underwent a 1.5-T or 3.0-T magnetic resonance imaging (MRI) scan of the affected knee to assess tear morphology, location and meniscus tissue quality. The mechanical Hip–Knee–Ankle angle (mHKA) was measured in all affected knees to evaluate coronal alignment. The severity of osteoarthritis was graded according to the Kellgren–Lawrence classification using standard anteroposterior and lateral radiographs. Postoperative meniscal healing was systematically assessed by MRI at 6 months. Clinical and functional outcomes were evaluated through standardized physical examinations and validated patient-reported outcome measures (PROMs), including the Knee Injury and Osteoarthritis Outcome Score (KOOS), the International Knee Documentation Committee (IKDC) Subjective Knee Evaluation Form, the Lysholm Knee Scoring Scale and the Tegner Activity Level Scale. PROMs were collected prospectively at baseline (preoperatively) and at final follow-up, with a mean follow-up duration of 2 years.

### 2.3. Surgical Technique

All procedures were performed arthroscopically by two senior orthopedic surgeons (G.Z. and K.G.P.) following a standardized surgical protocol. Patients were positioned supine on the operating table with a lateral thigh post and foot support, allowing for knee flexion up to 90°. A pneumatic tourniquet was applied to the proximal thigh and inflated after limb exsanguination. After local infiltration of the portal sites with lidocaine, standard anteromedial and anterolateral portals were established. A systematic diagnostic arthroscopy was then performed to assess the articular cartilage, ligamentous structures and both menisci. The meniscal tear was identified, debrided to healthy vascularized tissue using an arthroscopic shaver or rasp and subsequently mobilized. Meniscal repair was performed using an all-inside technique, an outside-in technique or a combination of both, depending on tear morphology, stability and vascular zone involvement (red-red or red-white zones). The all-inside technique was primarily used for posterior horn and mid-body tears with commercially available repair devices, whereas the outside-in technique was preferred for anterior horn tears and selected complex mid-body lesions using high-strength nonabsorbable sutures.

### 2.4. Postoperative Rehabilitation

A standardized postoperative rehabilitation protocol was applied to all patients. The operated limb was immobilized in a locked knee brace in full extension during rest for the first 2 weeks to protect the repair during the early healing phase. Active and passive range-of-motion (ROM) exercises were initiated immediately after surgery, with progressive knee flexion allowed under non-weight-bearing conditions. Patients used crutches and remained non-weight-bearing or touchdown weight-bearing for 4 to 6 weeks, depending on tear stability and repair complexity. Full weight-bearing was then gradually resumed according to clinical progression. Deep squatting, pivoting movements, and weight-bearing knee flexion beyond 90° were prohibited during the first 3 postoperative months.

### 2.5. Data Collection and Statistical Analysis

Demographic and clinical data were collected from electronic medical records and surgical registries, including age at surgery, sex, injury mechanism and chronicity, tear characteristics (type and location), repair techniques and implants used, secondary surgical procedures and clinical outcomes. Failure was defined as a subsequent surgical intervention involving partial meniscectomy or revision meniscal surgery. Clinical records and imaging studies were independently reviewed by two investigators (S.S. and M.D.). Discrepancies in radiographic grading or tear classification were resolved by consensus with the senior authors (K.G.P. and G.Z.). All data were subsequently verified by senior investigators (F.C. and F.P.) and interpreted collectively by the study team. Continuous variables were expressed as mean ± standard deviation (SD) or median with interquartile range (IQR), as appropriate, while categorical variables were reported as absolute frequencies and percentages. Preoperative and postoperative functional outcomes (KOOS, IKDC, Lysholm, and Tegner scores) were compared using the Wilcoxon signed-rank test due to the small sample size and non-normal distribution of the data. Effect sizes (Cohen’s d) with 95% confidence intervals were calculated for the main outcome measures to quantify the magnitude and precision of treatment effects. No formal survival or time-to-event analysis was performed given the limited sample size and number of events. Statistical significance was set at *p* < 0.05. All statistical analyses were performed using SPSS Statistics (version 26.0; IBM Corp., Armonk, NY, USA).

## 3. Results

### 3.1. Baseline Cohort Characteristics and Demographics

A total of 11 consecutive patients met the inclusion criteria and were included in the analysis. The cohort consisted of six women (54.5%) and five men (45.5%), with a mean age at surgery of 53.4 ± 4.1 years (range, 50–64 years). The mean clinical and radiological follow-up was 22.0 ± 16.8 months (range, 6–66 months). Nine patients (81.8%) underwent repair of a lateral meniscal tear, whereas two patients (18.2%) had a medial meniscal tear. Preoperative assessment of coronal alignment using weight-bearing long-leg radiographs demonstrated a mean mechanical hip–knee–ankle (mHKA) angle of 179.6° ± 2.4° (range, 175–184°), indicating overall neutral lower-limb alignment. Baseline radiographic osteoarthritis was limited, with a mean Kellgren–Lawrence (KL) grade of 1.0 ± 0.6 (range, 0–2). Four patients (36.4%) had KL grade 0, three (27.3%) had KL grade 1, and four (36.4%) had KL grade 2. Baseline demographic characteristics, clinical data, and individual mHKA measurements are reported in Table 1.

### 3.2. Patient-Reported Outcome Measures (PROMs)

**Figure 1:**
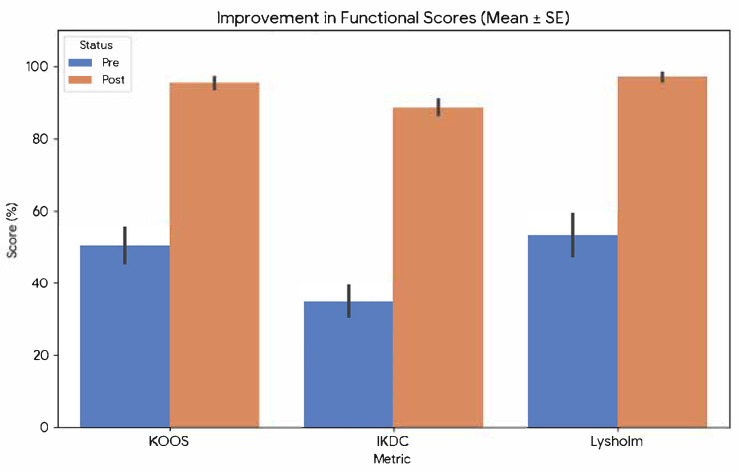
The bar chart illustrates the mean preoperative (blue) and postoperative (orange) functional scores for the KOOS, IKDC, and Lysholm metrics. Error bars represent the standard error (SE).

Analysis of patient-reported outcome measures demonstrated significant improvements from baseline to final follow-up across all evaluated scores. Preoperatively, patients showed marked functional impairment, with a mean Knee Injury and Osteoarthritis Outcome Score (KOOS) of 50.5% ± 16.0% (range, 16–70%), a mean International Knee Documentation Committee (IKDC) score of 43.0% ± 11.6% (range, 6.7–56.3%), and a mean Lysholm score of 53.4 ± 19.1 (range, 4–73). At final follow-up, all scores improved significantly (*p* < 0.001). The KOOS increased to 95.4% ± 5.1% (range, 88–100%), the IKDC score to 87.5% ± 8.3% (range, 77–100%), and the Lysholm score to 97.1 ± 3.5 (range, 94–100). The Tegner Activity Level Scale showed a median preoperative score of 6 (mean, 5.6 ± 1.0; range, 3–7), which was maintained at final follow-up (median, 6; *p* > 0.05). All patients returned to their pre-injury activity level without mechanical symptoms or subjective instability. A detailed summary of pre- and postoperative PROMs, including mean differences and confidence intervals, is provided in Table 2. Figure 1 illustrates the observed improvements in functional scores.

### 3.3. Complications, Failures, and Survival Analysis

Over the follow-up period, the overall clinical success rate for arthroscopic meniscal repair in this over-50 population was 81.81% (9 out of 11 patients). No major perioperative or systemic complications, such as deep vein thrombosis (DVT), superficial or deep surgical site infections or neurovascular injuries, were documented.

True construct failure occurred in two cases, both originating within the medial meniscal subgroup:

Complete Failure: One patient experienced acute symptom recurrence 8 months post-operatively due to a structural relapse of a bucket-handle tear in the medial meniscus, precipitated by an accidental low-energy trauma. This patient subsequently underwent a secondary arthroscopic revision procedure consisting of a partial medial meniscectomy.

Partial Failure: One patient reported persistent localized mechanical symptoms at 6 months postoperatively. Subsequent imaging and clinical evaluation demonstrated a partial failure of the suture construct at the posterior horn of the medial meniscus. The patient underwent secondary arthroscopic treatment consisting of a super-selective partial meniscectomy limited to the non-healed posterior horn segment. At final follow-up, complete symptom resolution and functional recovery were achieved in this patient. No additional secondary surgical interventions were recorded during the study period. 

All failures occurred in the medial meniscus subgroup. However, no statistically significant association was observed between coronal limb alignment (mHKA), tear morphology and meniscal healing or construct survivorship in this small cohort.

## 4. Discussion

The optimal management of meniscal pathology in middle-aged and master athlete populations remains debated in contemporary sports medicine. Meniscal deficiency is well known to disrupt knee biomechanical homeostasis, leading to altered contact mechanics, increased focal joint loading and progressive chondral degeneration with potential progression to osteoarthritis. While historical treatment paradigms favored resection, current trends increasingly emphasize joint-preserving strategies. Although several studies have reported favorable outcomes following arthroscopic meniscal repair in older patients, evidence remains mixed regarding its superiority over modern partial meniscectomy. A key challenge in this population is that most meniscal tears in patients aged 50 years and older are degenerative and often occur without a clear traumatic event. In this context, the ESSKA meniscus consensus statement (2016) recommends reserving partial meniscectomy for patients with persistent symptoms after a structured 3–6-month course of conservative management and in the absence of advanced osteoarthritis on plain radiographs [7]. While partial meniscectomy can provide reliable short-term pain relief and functional improvement, concerns remain regarding its long-term impact on joint degeneration due to loss of meniscal tissue. More recently, increasing attention has been directed toward meniscal preservation through arthroscopic repair in carefully selected patients over 50 years of age with preserved joint space and minimal chondral damage. This shift is supported by emerging evidence. Jaibaji et al. reported an overall failure rate of 15.6% in patients aged ≥40 years undergoing meniscal repair, with no significant differences across age groups and generally favorable long-term functional outcomes compared with resection [16]. Similarly, Everhart et al. reported an aggregate failure rate of 23% across 148 studies, independent of age [17]. Importantly, that analysis identified ACL deficiency, chronicity of injury, cartilage status, and tear morphology as more relevant predictors of failure than age alone [17]. Ventura et al. further demonstrated that although both repair and meniscectomy improve pain and function, meniscectomy may be associated with inferior long-term functional outcomes [18]. The findings of the present study are consistent with this literature. We observed significant improvements in KOOS, IKDC, Lysholm, and Tegner scores following arthroscopic meniscal repair in a carefully selected cohort of patients over 50 years of age. The overall failure rate was 18%, which falls within previously reported ranges [16,17]. Notably, medial meniscal tears appeared to be associated with a higher proportion of failures in this cohort, with all failures occurring in medial lesions. In particular, bucket-handle tears of the medial meniscus in first-time injury settings may represent a potential risk factor for repair failure, although this finding should be interpreted cautiously given the small sample size. This may reflect the technical and biological challenges of repairing complex medial meniscal lesions in older patients. Lower limb coronal alignment is an important consideration when selecting candidates for meniscal preservation procedures, as malalignment may increase compartmental loading and influence both tear development and healing potential. In our cohort, lateral meniscal tears were observed in patients with mHKA values ranging from 175° to 184°, with valgus alignment present in 66.7% of cases. Medial meniscal tears were observed in patients with mHKA values of 178° and 176°, consistent with varus alignment in all cases. However, no definitive association between alignment, tear morphology and repair failure could be demonstrated in this small sample. Overall, our surgical approach was guided by the principle of preserving native meniscal tissue whenever feasible to reduce peak contact stresses and protect the articular cartilage. This strategy should, however, be applied selectively in well-aligned, active patients with preserved joint compartments and limited degenerative changes.

### Limitations

Despite the promising functional results, several inherent limitations of this study must be acknowledged. First, the retrospective design may have introduced selection and information bias. Furthermore, this was a single-center study, potentially limiting the generalizability of the findings. The small sample size (n = 11) of active patients may restrict the external validity of our findings, preventing direct generalization to the broader, more sedentary population over the age of 50 presenting with degenerative meniscal pathology. Second, although arthroscopic partial meniscectomy represents the historical and primary treatment modality for degenerative tears following the failure of conservative management, our study design lacked a concurrent, randomized control group for comparative efficacy analysis. In addition, follow-up duration was variable among patients. Finally, while tear morphology and lesion dimensions were systematically recorded during the index diagnostic arthroscopy, they were not comprehensively cross-tabulated in the final statistical model in order to minimize confounding variables and avoid over-stratifying our small cohort. Although both failures occurred in the medial meniscus subgroup, factors such as tear morphology, vascularity, and subtle alignment-related biomechanical differences may have contributed to these outcomes; however, the limited number of failures precludes any definitive conclusions. Future prospective, randomized trials with larger sample sizes and long-term radiological tracking are warranted to fully delineate the survivorship boundaries of meniscal repair in master athletes. Moreover, the limited sample size and the markedly unbalanced distribution between lateral and medial meniscal lesions prevented adequately powered subgroup analyses. Consequently, comparisons according to tear location should be regarded as exploratory and interpreted with caution rather than as definitive evidence of differential outcomes.

## 5. Conclusions

In this small cohort, meniscal repair with suture was associated with substantial improvements in knee function and return to pre-injury activity levels in 81.8% of patients. One complete failure and one partial failure were observed, both occurring in patients with medial meniscal tears. Meniscal tears in middle-aged patients remain challenging to manage, particularly in active individuals and in cases following failed conservative treatment, which is generally considered the first-line approach for degenerative lesions. Current evidence supports a more individualized, patient-specific approach, suggesting that chronological age alone should not be considered an absolute contraindication to meniscal repair. Preoperative assessment with weight-bearing long-leg and standard radiographs is essential to evaluate osteoarthritis and coronal alignment, which may influence treatment strategy and the potential need for concomitant corrective osteotomy. Future randomized controlled studies with larger sample sizes and longer follow-up are required to confirm these findings and further clarify optimal indications for meniscal repair in this population. Overall, our findings suggest that in carefully selected middle-aged active patients with preserved limb alignment and low-grade osteoarthritis, meniscal repair may represent a viable joint-preserving option, particularly for lateral meniscal lesions. However, these results should be interpreted as preliminary due to the limited sample size.

## Figures and Tables

**Table 1:** Demographic, Kellgren and Lawrence (KL) values, Mechanical Hip–Knee–Angle values and site of meniscus lesion. Patients identified in **bold and underlined** text correspond to cases of clinical failure, defined by the necessity for reoperation.

Patient	Sex	Age	FU *	KL	HKA	SITE
Patient 1	F	64	34	1	183	LM
Patient 2	M	50	30	1	182	LM
Patient 3	F	51	16	2	184	LM
Patient 4	M	51	8	1	175	LM
Patient 5	F	52	18	1	176	LM
**Patient 6**	**F**	**50**	**13**	**0**	**178**	**MM**
Patient 7	M	54	16	1	180	LM
Patient 8	M	53	21	2	177	LM
Patient 9	F	50	66	1	183	LM
Patient 10	F	50	6	1	182	LM
**Patient 11**	**M**	**50**	**14**	**1**	**176**	**MM**

* Months of follow-up.

**Table 2:** Results for all PROMs (KOOS, IKDC, Lysholm, Tegner).

Patient	KOOS		IKDC		Lysholm		Tegner	
	Pre	Post	Pre	Post	Pre	Post	Pre	Post
Patient 1	37	100	19.5	89.7	47	100	5	5
Patient 2	16	90	6.7	77	4	95	3	3
Patient 3	41	92	36.8	92	42	95	5	5
Patient 4	52	99	44.8	94.3	65	90	7	7
Patient 5	56	100	41.4	92	47	94	6	6
Patient 6	52	99	37.9	87.4	65	100	6	6
Patient 7	47	100	24.1	90.8	58	100	5	5
Patient 8	68	88	37.9	78.2	73	100	6	6
Patient 9	70	100	56.3	100	63	100	6	6
Patient 10	69	90	48.3	83.9	69	99	6	6
Patient 11	47	91	29.9	89.7	54	95	7	7
Mean	50.45	95.36 *	43	87.54 *	53.35	97.09 *	5.64	5.64 *
Change	+44.91		+44.54		+43.74		0	

* = statistically significant increase (*p* < 0.05).

## Data Availability

The data presented in this study are available on reasonable request from the corresponding author due to privacy and ethical restrictions.
